# HDAC Regulates Transcription at the Outset of Axolotl Tail Regeneration

**DOI:** 10.1038/s41598-019-43230-6

**Published:** 2019-05-01

**Authors:** S. Randal Voss, Larissa V. Ponomareva, Varun B. Dwaraka, Kaitlin E. Pardue, Nour W. Al Haj Baddar, A. Katherine Rodgers, M. Ryan Woodcock, Qingchao Qiu, Anne Crowner, Dana Blichmann, Shivam Khatri, Jon S. Thorson

**Affiliations:** 1 Department of Neuroscience, Spinal Cord and Brain Injury Research Center, and Ambystoma Genetic Stock Center, University of Kentucky, Lexington, KY 40506 USA; 2 College of Pharmacy and Center for Pharmaceutical Research and Innovation, University of Kentucky, Lexington, KY 40536 USA; 3 Department of Biology, Keene State College, Keene, NH 03431 USA

**Keywords:** Chemical genetics, Regeneration, Developmental biology

## Abstract

Tissue regeneration is associated with complex changes in gene expression and post-translational modifications of proteins, including transcription factors and histones that comprise chromatin. We tested 172 compounds designed to target epigenetic mechanisms in an axolotl (*Ambystoma mexicanum*) embryo tail regeneration assay. A relatively large number of compounds (N = 55) inhibited tail regeneration, including 18 histone deacetylase inhibitors (HDACi). In particular, romidepsin, an FDA-approved anticancer drug, potently inhibited tail regeneration when embryos were treated continuously for 7 days. Additional experiments revealed that romidepsin acted within a very narrow, post-injury window. Romidepsin treatment for only 1-minute post amputation inhibited regeneration through the first 7 days, however after this time, regeneration commenced with variable outgrowth of tailfin tissue and abnormal patterning. Microarray analysis showed that romidepsin altered early, transcriptional responses at 3 and 6-hour post-amputation, especially targeting genes that are implicated in tumor cell death, as well as genes that function in the regulation of transcription, cell differentiation, cell proliferation, pattern specification, and tissue morphogenesis. Our results show that HDAC activity is required at the time of tail amputation to regulate the initial transcriptional response to injury and regeneration.

## Introduction

Wound healing processes are rapidly activated after injury to prevent infection and repair damaged tissues^1^. In contrast to mammals, highly regenerative salamanders repair damaged tissues without scarring and are capable of regenerating appendages and organs^2^. While many aspects of wound healing are conserved among organisms, critical differences in early wound healing processes may explain scar-free healing and competence for regeneration^3^. Open wounds in salamanders are rapidly closed by epidermal cells that migrate from the basal layer of the epidermis^4^. Macrophages infiltrate the injury site^5^ and presumably release molecules that prevent infection, including reactive oxygen species (ROS) and antibacterial proteins. These cells clear the injury site of debris, and dying and senescent cells^6^, change the nature of the extracellular matrix, and release cytokines that induce cellular and molecular changes among infiltrating and surviving cells, including changes in gene expression. The diversity of the early transcriptional response suggests that regeneration in salamanders requires simultaneous expression of wound-healing and developmental genes^7,8^.

Although there have been few studies to date [but see^9^], injury cues likely act through epigenetic mechanisms that alter chromatin structure and properties of proteins that function in transcriptional regulation and cell signaling^10^. These epigenetic mechanisms include DNA methylation and various chemical modifications of histones, including acetylation-deacetylation [reviewed by^11^]. Regions of the genome with high levels of DNA methylation and/or low levels of histone acetylation are characterized by compacted chromatin and transcriptional repression, while low levels of DNA methylation and/or high levels of DNA acetylation are characterized by open chromatin and transcriptional activation. Because genomic data have been lacking until recently^12,13^, few studies have examined epigenetic mechanisms during salamander regeneration. Yakushiji *et al*.^14^ found that *sonic hedgehog* (*shh*), an essential limb patterning gene, is expressed during limb regeneration in regeneration competent Xenopus tadpoles and axolotls, but not during stages of development when Xenopus are refractory to regeneration. Expression of *shh* was correlated with a lower methylation status of the *shh* promoter region and histone modifications^15^, thus implicating epigenetic regulation. Building on this work, Hayashi *et al*.^16^ showed that histone modifications are important for regulating genes that maintain intrinsic limb-cell identities during Xenopus limb bud regeneration. Aguilar and Gardiner^17^ examined transcriptional changes of *dnmt3* during axolotl limb regeneration, a methyltransferase that mediates *de novo* methylation of cytosine residues in DNA. They found that nerve signaling transiently downregulated *dnmt3* in basal keratinocytes of the early wound epidermis, suggesting a decrease in methylation status and an associated de-repression of genes (e.g. *sp9*) necessary for the formation of a specialized wound epidermis that is required for blastema formation. Taylor and Beck^18^ used valproic acid (VPA) to reduce histone deacetylase (HDAC) activity and inhibit tail regeneration in Xenopus and axolotl embryos. Interestingly, they found that pre-treatment of Xenopus embryos with VPA prior to tail amputation did not inhibit regeneration, nor did VPA treatment administered after 6-hours post amputation (6 hpa). Their results suggested that HDAC activity is required sometime within the first 6 hours of injury to trigger the re-expression of developmental genes, although no transcriptional data were presented to support this hypothesis. Tseng *et al*.^19^ also used VPA and trichostatin A to inhibit Xenopus tadpole tail regeneration. They observed spatially aberrant expression of developmental genes (at 24 hpa) that are required for tail regeneration (*notch1, bmp2*), consistent with the idea that HDAC activity is required during regeneration to regulate gene expression. While these few studies show that DNA methylation and HDAC activity are essential for amphibian appendage regeneration, much remains to be learned about these and other mechanisms of epigenetic regulation.

Using highly regenerative axolotl embryos, we performed a chemical genetic screen using commercially available compounds that target protein activities associated with epigenetic regulation. We identified 55 chemicals that inhibited tail regeneration at 7-days post amputation (7 dpa), with the highest frequency of hits associated with histone deacetylase inhibitors. Focusing on a specific HDAC class I inhibitor, we show that 10 μM romidepsin potently inhibits tail regeneration at 7 dpa when only treating embryos for 1-minute post amputation (1 mpa). Romidepsin-treated embryos did commence regeneration after 7 dpa, however the resulting tails and especially the tailfins were abnormally patterned. Using microarray analysis, we show that romidepsin altered transcription at two early post-amputation time points (3 and 6 hpa), with a high number of affected genes predicted to function in transcriptional regulation. Our results show that HDAC activity is required at the time of tail amputation to regulate the initial transcriptional response to injury and regeneration.

## Results

### Chemical Screen of Epigenetic Compounds

**Figure 1:**
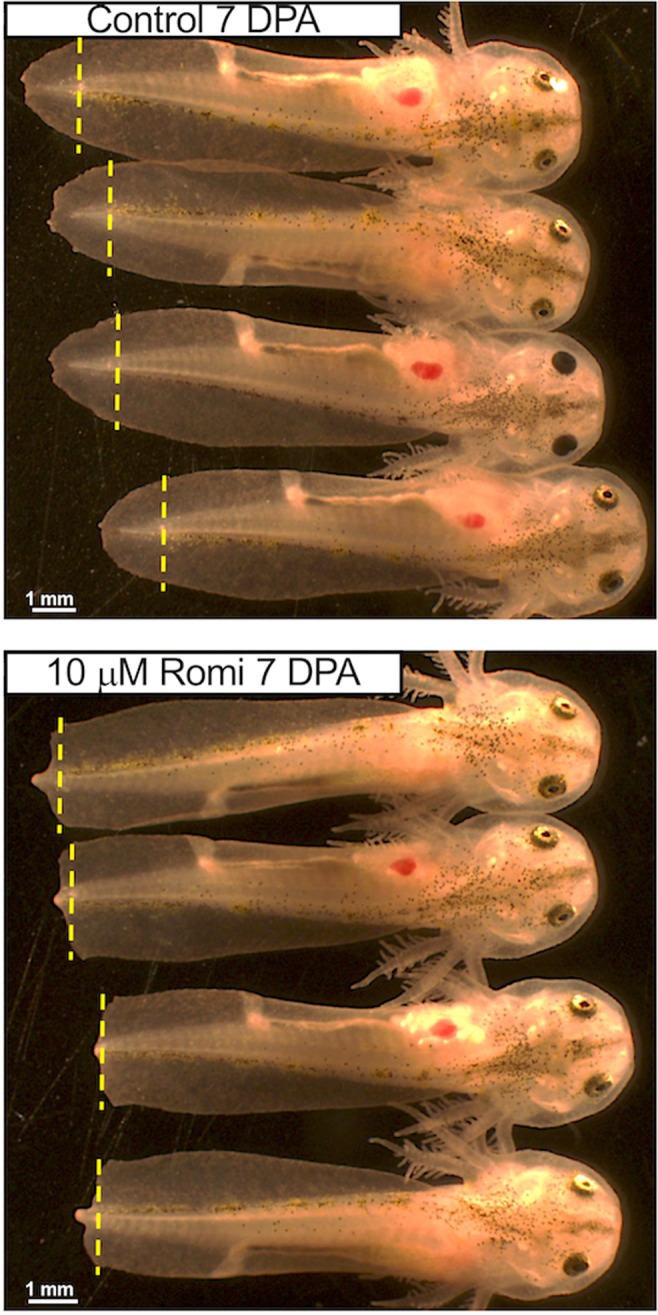
Romidepsin (Romi) and other class I and class II HDACi yielded a similar tail morphology at 7 dpa, consistent with inhibition of regeneration. The vertical, yellow dashed lines show where tails were amputated.

Amputations were performed on axolotl embryos to remove 2 mm of distal tail tissue. Immediately after tail amputation, embryos were reared in the presence of chemicals (N = 172) that target epigenetic mechanisms (Supplementary Table 1). Seven-day post amputation embryos were scored for survival and the survivors were photographed. Typically, tail regeneration is completed in 7 days and at this time the regenerated tail tip approximates the shape of the pre-amputated tail (see control in Fig. 1). Six different deviations from a normally patterned tail were observed and classified as inhibitory outcomes (Supplementary File 1; Supplementary Table 1). A total of 38 chemicals yielded inhibitory outcomes for 3–4 replicate embryos in two separate trials at 10 μM; these chemicals were considered to reproducibly inhibit regeneration. No chemicals were found to cause more tissue growth than is typical of tail regeneration and 23 chemicals caused the mortality of >2 embryos and were considered toxic. Of the remaining chemicals, we note that 17 chemicals that were scored non-inhibitory at 10 μM were found to be inhibitory at 20 μM. While the remaining chemicals were scored as non-inhibitory, they may prove to be valuable research tools with further optimization of dose. Eighteen of the regeneration inhibitory compounds antagonistically target HDACs and 8 antagonistically target bromodomains of epigenetic reader proteins (BET, SMARCA, and BRPF1 families). The remaining compounds antagonistically target JAK1/2 (N = 8), aurora kinase (N = 6), poly ADP ribose polymerase (N = 3), histone methyltransferases (N = 4), a sirtuin, and a lysine specific histone demethylase. We note that some of these chemicals have pleiotropic effects beyond the mechanisms that are listed above.

The HDAC inhibitors yielded a similar 7 dpa tail phenotype in embryos, characterized by either a small protuberance of somite tissue or no somite tissue at the position of the midline, and no or little tailfin outgrowth (Fig. 1). The commonality of this phenotype among HDACi-treated embryos suggested inhibition of a similar regeneration mechanism. Indeed, the HDACi identified from the screen target class I and II HDACs with varying specificity. HDACi that inhibit other HDAC classes did not pass the criteria of complete inhibition of regeneration. This included VPA, which was previously reported to quantitatively inhibit axolotl tail regeneration^18^. Given the high hit rate for HDACi from our screen, we focused subsequent experiments on romidepsin and belinostat, two FDA approved anti-cancer drugs^20,21^.

### Initial Microarray Analysis of HDACi Treatment

**Figure 2:**
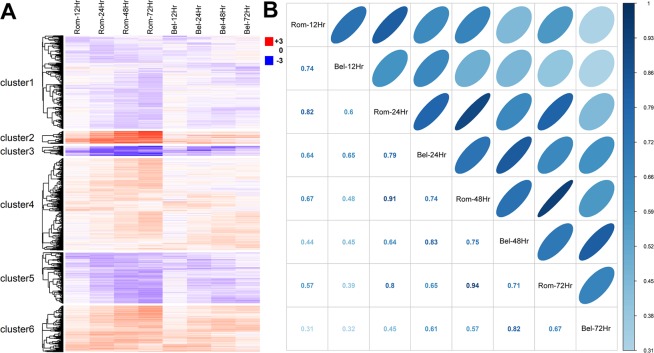
(**A**) Differentially expressed probe sets (N = 6,540) were clustered hierarchically using Pearson correlation as a distance metric. (**B**) The correlation of average fold change between romidepsin and belinostat samples. The magnitude of the correlation coefficients is represented by the intensity of blue, dark being highly correlated, and by the shape of ellipses, with narrow being highly correlated. These panels show that rombidepsin and belinostat induced similar, directional changes in gene expression but the magnitude of the expression differential (relative to controls) was greater for romidepsin-treated embryos.

A microarray analysis was performed to identify changes in gene expression that would provide mechanistic insights about HDACi inhibition of regeneration. Estimates of gene expression were compared between treated (romidepsin and belinostat) and untreated control embryos at the time of amputation, and then at 12, 24, 48, and 72 hpa. Genes were identified as significantly differently expressed if they met statistical (moderated t-test, FDR <0.05) and fold change criteria (1.5-fold difference) in relation to control embryos (Supplementary Table 2). For each post-amputation time point analyzed, more significant genes were identified for romidepsin-treated embryos than for belinostat-treated embryos. For example, at 12 hpa 1275 and 1015 genes were identified as significant for romidepsin and belinostat treated embryos, respectively. More significant genes were identified at later post-amputation time points for romidepsin treated embryos, with 4584 genes identified at 72 hpa. In contrast, the number of significant genes plateaued at 48 hpa for belinostat treated embryos; still, 1442 genes were identified as significant at 72 hpa. Thus, while both HDACi profoundly altered gene expression, romidepsin affected more genes. However, we note that genes identified as significant in the romidepsin treatment, but not the belinostat treatment, mostly exhibited correlated patterns of expression, as did genes that were identified as significant in the belinostat treatment, but not the romidepsin treatment (Fig. 2). This suggests that romidepsin and belinostat affect transcription of the same gene targets, but romidepesin is more potent in affecting transcriptional output at the concentration tested. Thus, while romidepsin and belinostat induced similar, directional changes in gene expression during axolotl tail regeneration, the magnitude of gene expression change was greater for romidepsin-treated embryos. This reveals an interesting facet of HDAC regulation of gene expression in the axolotl embryo model– it is potentially dose dependent and titratable using HDACi.

### Temporal Analysis of HDACi Treatment

**Figure 3:**
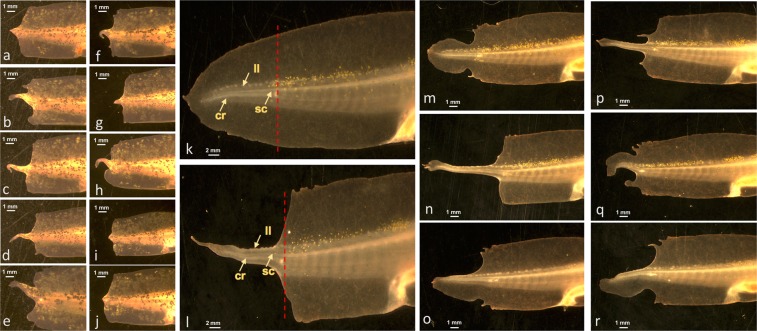
Examples of tail regeneration at 21 dpa. (**a–e**) Individuals that were treated with 10 μM romidepsin for 3 hpa. (**f**–**j**) Individuals that were treated with 10 μm romidepsin for 24 hpa. (**k**) An individual that was not treated with romidepsin. The arrows indicate the spinal cord (sc), lateral line (ll), and cartilaginous rod in the tail regenerate. The red hatched-line shows the position of the amputation plane. (**l**–**r**) Individuals that were treated with 10 μm romidepsin for 1 mpa.

In the initial chemical screen, embryos were treated continuously with compounds for 7 dpa. To determine the critical window within which romidepsin inhibits regeneration, embryos were treated for varying lengths of time post-amputation (Supplementary Table 3). By systematically decreasing the treatment time, we found that a 1 mpa treatment with 10 μM romidepsin (but not 10 μM belinostat) reproducibly yielded a non-regenerative phenotype at 7 dpa. We note that a 0.5 uM romidepsin treatment did not inhibit regeneration, nor did a 10 uM romidepsin pre-treatment for 24 hrs prior to amputation. Thus, the inhibitory effect of romidepsin coincided with tissue injury and required a dose ≥1.0 μM. Interestingly, the length of time that embryos were treated with romidepsin (1 mpa-7 dpa) did affect regenerative potential after 7 dpa. Regeneration of tail fin tissue was variable and abnormal, with less tissue regeneration and more extreme patterning defects associated with longer post-amputation treatment times. For example, individuals that were treated for only 1 mpa regenerated more tissue by 21 dpa than individuals that were treated continuously for 3 or 24 hpa (Fig. 3). In particular, individuals that were only treated for 1 mpa exhibited more tailfin regeneration and outgrowths of tailfin tissue along the distal tail tip. Although 1 mpa treated embryos exhibited abnormal tail patterning, the length of regenerated spinal cord within these tails was not different from control embryos at 21 dpa (N = 12; Student’s t-test, p = 0.74). These results suggest that romidepsin specifically affected tailfin outgrowth and tailfin outgrowth is necessary for spinal cord regeneration.

### Romidepsin treatment from 0–3 hpa significantly affected gene expression at 3 and 6 hpa

**Figure 4:**
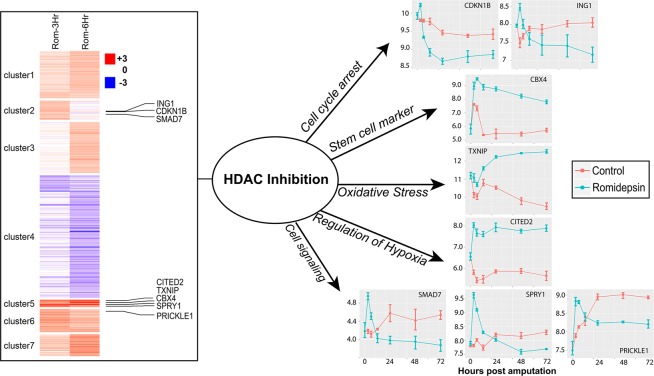
Hierarchical clustering of probe sets (N = 482) expressed differentially at 3 and 6 hpa between romidepsin-treated and control embryos. Expression profiles are shown for a few of the many regulatory genes that were discovered to be differentially expressed.

Given the large number of genes identified as differentially expressed at 12 hpa and the result that 1 mpa treatment with romidepsin inhibited tail regeneration, we performed a second microarray analysis of two earlier, post-injury time points – 3 and 6 hpa. Using statistical and fold change criteria, a total of 128 and 239 probesets detected significantly higher gene expression in romidepsin-treated embryos at 3 and 6 hpa respectively, while 56 and 191 probesets detected significantly higher gene expression in control embryos at 3 and 6 hpa respectively. (Supplementary Table 4; Fig. 4). Thus, consistent with the first microarray experiment, the number of significant genes discovered increased with post-amputation time. There was considerable overlap among the genes identified at 3 and 6 hpa. Of the 184 significant genes identified at 3 hpa, 132 were also identified as significant at 6 hpa and all of these genes showed the same directional change. Furthermore, preliminary analyses showed that shared versus unique genes between the 3 and 6 hpa lists tended to enrich the same gene ontology terms. Thus, all non-redundant genes that were identified as significant at either 3 or 6 hpa were used for gene ontology enrichment analysis using Panther^22^. Genes that were expressed (on average) more highly in control embryos enriched relatively few Gene Ontology (GO) terms associated with the regulation of metabolism, mRNA processing, and oxygen binding (Supplementary Table 5). We also note that 33 of these genes (*agxt2l1, calhm1, ccnb1, coq10b, ctgf, ctsl2, cyp26a1, cyp26b1, dnajc10, fbxo5, has2, hpx, klf10, krt17, lep, lypd6, mas1, mmp1, mmp19, mmp2, mmp3, nfil3, nov, pdlim7, phlda2, pthlh, r3hdml, rnf24, socs1, tgif1, tmem92*) were identified as differently expressed 24–168 dpa when blocking WNT signaling and tail regeneration using this same tail amputation assay^23^. This strongly suggests that HDAC activity is associated with proper Wnt signaling and the transcriptional regulation of key regeneration genes. For example, *leptin* (*lep*) is highly expressed in regenerating hearts and fins of zebrafish and may act as a general trigger of tissue regeneration^24^. Of the genes that were expressed more highly in control embryos, *lep* exhibited the greatest expression difference between control and treated embryos.

A large number of gene ontology terms were significantly enriched by genes that were more highly expressed in romidepsin-treated embryos. In fact, the number of gene ontology terms that were identified (N = 298) exceeded the number of genes that were submitted for the enrichment analysis (N = 242). This result is explained by the very large number of transcription factors and signaling molecules in the gene list, and the diverse roles the genes play in regulating RNA polymerase II mediated transcription. For example, 110 genes that were more highly expressed in romidepsin-treated embryos encode proteins that positively or negatively regulate gene expression (Table 1). These genes regulate diverse biological processes including cell differentiation, cell proliferation, pattern specification, and tissue morphogenesis. Overall, romidepsin altered the expression of key transcriptional regulators, signaling factors, and patterning molecules, thus implicating HDAC as an important regulator of regeneration-associated transcriptional responses immediately after injury.

**Table 1:** Genes (N = 110) associated with transcriptional regulation that were expressed more highly in romidepsin-treated embryos at 3 or 6 hours-post amputation.

arid4a	elf3	ino80b	mxi1	sfrs17a
atmin	emx1	insig1	notch1	sgk1
bc11b	emx2	insm1	nr2f1	smad7
bcor	en2	irf1	nr2f2	sostdc1
btg1	fam46C	jag1	nr4a1	sox8
c14orf4	fos	jag2	nrarp	sp7
casz1	foxc1	jund	onecut2	spen
cbx4	fzd8	klf4	osr1	tbx15
cbx8	gtd2b	klf5	ovol2	tfap2a
cby1	hes5	lbh	pck1	tmem100
cdc6	hey1	lefty1	per1	trak1
cdkn1b	hoxa3	lmx1b	pkp1	tsc22d3
cited2	hoxb9	lrrc14	pnrc1	tshz1
cyr61	hoxc10	mafb	prickle1	txnip
dact1	hoxc8	maml2	rara	znf250
ddit3	hspa8	med7	rbm24	znf281
dll1	htf4	meis1	rbm38	znf300
dlx6	id1	meis2	rg9mtd1	znf510
dnajb5	id2	mn1	rgma	znf703
ebf2	ift57	myc	rgmb	znf750
ebf3	ing1	mycn	ror2	znf777
efna1	ing2	myf5	sfrp2	znf821

### Romidepsin treatment from 0–1 mpa significantly affected gene expression at 3 hpa

To further validate effects of romidepsin on gene expression, we treated embryos for 1 mpa with romidepsin and used microarray analysis to test for gene expression differences at 3 hpa. We found that 208 of the 227 genes identified as significantly differentially expressed when treated with romidepsin for 0–3 hpa, also exhibited a statistically significant difference at 3 hpa when only treated for 1 mpa (Supplementary Table 4). Moreover, the correlation of fold change for these genes between the 0–3 hpa and 0–1 mpa treatments was r = 0.94. These results show that romidepsin potently, rapidly, and reproducibly affected gene expression at the time of amputation injury.

### Analysis of dividing cells

**Figure 5:**
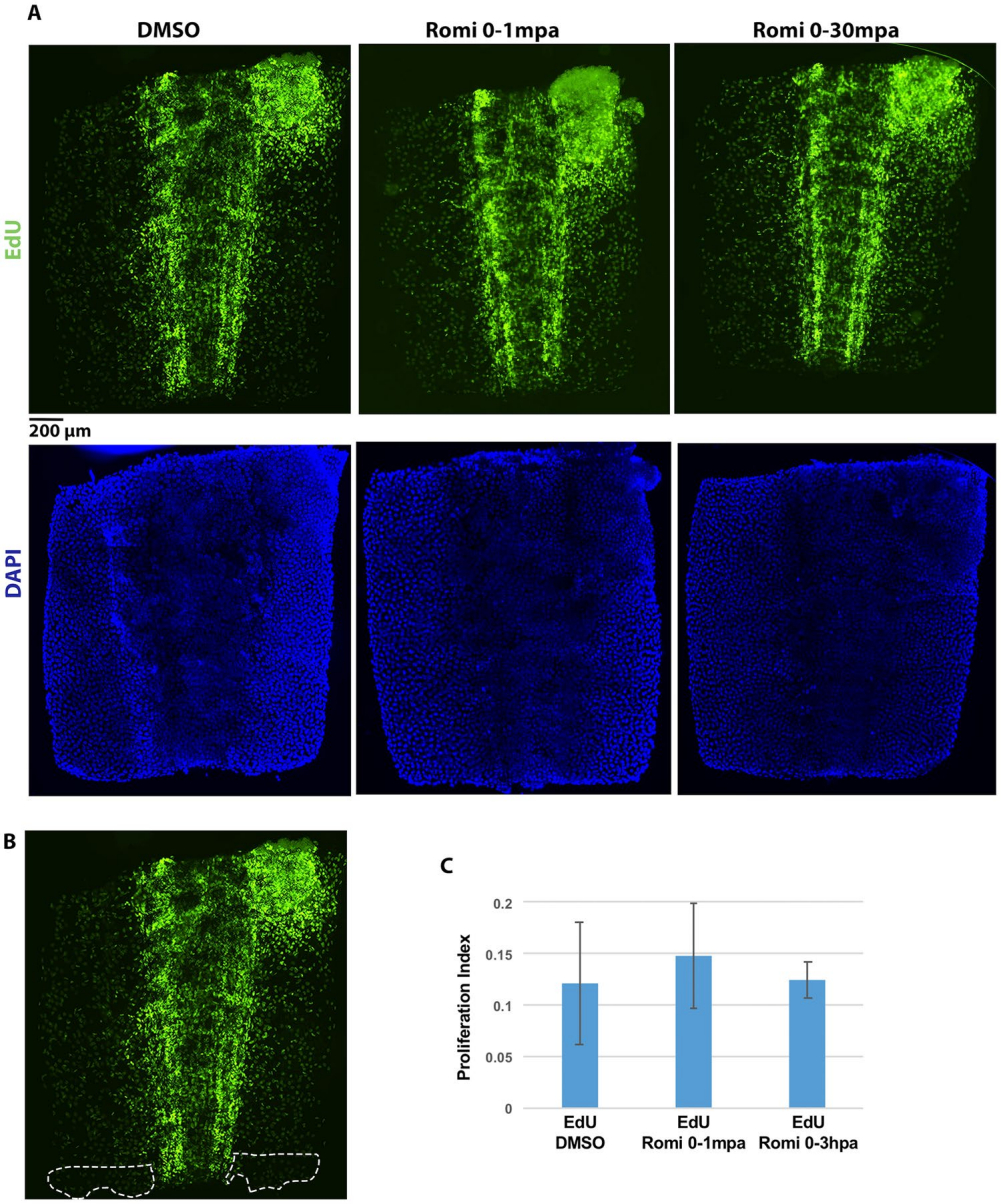
Romidepsin treatment does not affect cell proliferation at 3 hpa. (**A**) Embryo tail images showing EdU and DAPI staining at 3 hpa for control (DMSO) and treatment groups (Romi 0–1 mpa, and Rmoi 0–3 hpa). Scale bar = 200 μ. (**B**) Representative image for the 200 μ area of the tail tip used for cell counts and calculations. (**C**) EdU quantification for embryos treated with DMSO (N = 6) and Romidepsin for 0–1mpa (N = 6) and 0–3 hpa (N = 6). Error bars are standard deviations of the mean.

Romidepsin is an FDA approved anticancer drug that blocks cell cycle progression in some types of tumor. To determine if romidepsin similarly affects cells during axolotl tail regeneration, we quantified and compared the number of dividing cells between control and romidepsin-treated embryos at 3 hpa. Dividing cells were identified by EdU, a thymidine analogue which is incorporated into DNA during S-phase. Many EdU positive cells were observed, indicating abundant cell proliferation in these developing embryos and no global effect of romidepsin on cell cycle entry (Fig. 5A). The number of EdU positive cells within 200 μm of the amputation plane did not differ significantly between control and romidepsin-treated embryos (Student’s t-test. p = 0.56), and there were no apparent differences for other regions of the tail (Fig. 5B,C). Thus, while acute romidepsin treatment inhibited tail regeneration at 7 dpa, it did not significantly decrease the number of dividing cells at an early post-amputation time point.

## Discussion

We performed a chemical genetic screen of amphibian appendage regeneration focusing on chemicals that modify epigenetic mechanisms. Fifty-five chemicals were found to inhibit axolotl tail regeneration, with HDACi represented highly among this group. HDACs remove acetyl groups from lysine amino acid residues that comprise proteins [reviewed by^25^]. HDACs target a diversity of proteins but are best known for the removal of acetyl groups from lysine tails of histones. Histone deacetylation relaxes the structure of chromatin and makes DNA more accessible to proteins and RNAs that regulate gene expression. Typically, HDAC activity is associated with the activation of gene expression, however deacetylation of regulatory proteins can activate or repress transcription. We also identified 8 compounds that antagonistically target bromodomain-containing proteins that act as readers of ε-N-lysine acetylation marks that affect transcriptional regulation [reviewed by^26^]. Thus, 47% of the chemicals that were identified in our screen (26 of 55) are associated with epigenetic writing and reading of lysine acetylation, and both mechanisms are known to affect the transcriptional regulation of similar gene targets. If a study of bromodomain inhibitors using the axolotl embryo model yielded parallel results, it would further establish that cancer and tissue regeneration share mechanisms of epigenetic regulation.

In cancer studies where bromodomain and HDAC inhibitors have been studied most, both mechanisms have been shown to increase the expression of *cdkn1b* and *txnip* in tumor cells to induce cell cycle arrest^27–30^. Accordingly, there is great interest in developing bromodomain and HDAC inhibitors as combinatorial therapeutics as these compounds target similar genes and elicit similar biological effects^31^. We similarly observed increased expression of *cdkn1b* and *txnip* in romidepsin-treated embryos, but did not observe fewer dividing cells relative to controls at 3 hpa, as would be expected if romidepsin induced cell cycle arrest. However, if romidepsin only affected cell cycle entry of a small number of cells necessary for regeneration, it would be difficult to identify these against the backdrop of abundant cell proliferation in rapidly developing embryos. It would similarly be difficult to identify changes in cellular differentiation and cell death for a small number of essential cells. Interestingly, Luchenko *et al*.^32^ found that romidepsin similarly induced histone acetylation across 18 different human cell lines, but not cell death. They speculated that only cells primed for apoptosis undergo cell death in response to global changes in acetylation. It will be important in future studies to identify the cells that are affected by romidepsin treatment and determine if the effects of romidepsin are cell type and/or cell context specific during axolotl tail regeneration.

In addition to genes that are typically dysregulated in cancer cells after HDACi treatment, we observed changes in axolotl genes that regulate transcription and developmental signaling pathways (*e.g. smad7, bmp2, id1, id2, notch1, jag1, dll1, prickle1, dact1, myc, mycn, ngfr, hes5, meis2, sox8, foxc1, lefty1, spry1*). Transcriptional changes that alter the wound-healing environment or fundamental signaling pathways might induce changes in progenitor cells that are non-permissive for regeneration. For example, gene expression changes suggest that TGFβ signaling was attenuated. Smad7, a negative inhibitor of smad2/3 signaling, was up-regulated at 3 hpa in romidepsin-treated axolotls, while *klf10*, a negative inhibitor of *smad7*, was down-regulated. Also, inhibitors of canonical (*dact1*) and non-canonical Wnt signaling, (*prickle1)*, TGFβ signaling (*smad7*), BMP signaling (*sostdc1)*, and FGF signaling (*spry1*) were also upregulated at 3 hpa. All of these pathways are essential for regeneration of tail appendages in amphibians and zebrafish^23,33–35^. We also observed changes in the expression of genes that function in proximal-distal patterning during appendage development and regeneration. Genes implicated in retinoic acid (RA) metabolism and signaling (*abca1, aqp3, cyp26a1, cyp26b1, dhrs3, ezh2, fgfr2, klf4, lep, meis1, meis2, osr1, pck1, rdh10*), which are typically associated with proximal patterning of anterior-posterior morphogenetic fields [reviewed by^36^], were dysregulated in romidepsin-treated embryos; and homeotic genes (*hoxa3, hoxb9, hoxc8, hoxc10*) that define medial spatial positions of the developing vertebrate body axis were upregulated. These expression patterns suggest that HDACi activated proximal patterning genes in the distal tip of the regenerating tail, as this is where tissue was collected for gene expression analyses.

Although axolotl embryos treated with romidepsin for 1 mpa did not show signs of tissue regeneration at 7 dpa, they subsequently regenerated abnormally patterned tails. Because these individuals were capable of regenerating distal tailfin tissue, other tissues within the tail also regenerated. For example, the amount of spinal cord tissue that regenerated between control and 1 mpa romidepsin treated embryos was not significantly different at 21 dpa, even though the tails of the later were abnormally patterned. These results suggest that romidepsin affects progenitor cells in the tailfin mesenchyme, perhaps by arresting growth, or by inducing death or differentiation. It is possible that the 1 mpa treatment was not sufficiently long to affect all of these cells and thus tailfin regeneration was delayed and not completely inhibited at 21 dpa. The small outgrowths of tailfin along the distal margin of the tails is supportive of this idea as these regions may contain progenitor cells that were not affected by romidepsin treatment, and within these foci became nested with sufficient proximal-distal patterning information to facilitate local outgrowths.

The temporal regulation of transcription factor complexes at the time of injury is probably essential for regeneration. Presumably, the genes that were activated at 3 and 6 hpa in romidepsin-treated embryos are normally repressed by HDAC activity after injury. Consistent with this inference, *hdac1* expression was approximately 8–9 at the time of amputation, which in terms of Affymetrix log2 normalized data, is a relatively high expression level. This suggests that class I HDAC activity is maintained at appreciable homeostatic levels within appendages to repress genes that would disrupt initial injury responses and the onset of regeneration transcriptional programs. This is consistent with the results of Tseng *et al*.^19^ who observed mis-expression of *notch1* and *bmp2* after HDACi treatment of amputated Xenopus tadpole tails at 24 hpa. In addition to these two genes, we identified multiple Notch (*bhlhb2*, *cdkn1b, dll1, foxc1, hey1, hes5, jag1, jag2, krt19, lfng, mint/spen, myc, nov, nrarp, c8orf4, ovol2, rbm15, tmem100*) and BMP (*ctsl2, col2a1, cyr61, hes5, id1, id2, rgma, rgmb, ror2, sfrp2, sostdc1, smad7*) pathway genes and gene targets that were dysregulated by romidepsin. Overall, many transcription factors, chromatin remodeling genes, and regulators of signaling pathways (e.g. Tgfβ, Wnt, FGF, BMP, Notch, and RA) were differentially expressed at 3 hpa in romidepsin-treated embryos. HDAC activity at the time of tissue injury is critical for regulating an initial transcriptional response to injury that leads to a successful regenerative outcome.

HDAC-containing corepressor complexes are known to regulate the timing of transcription-mediated developmental events in anuran amphibians. For example, HDACs interact with thyroid hormone receptors (TRs) to regulate the proper timing of metamorphic transcriptional programs among different tissues and organs [reviewed by^37^]. Metamorphosis in amphibians is initiated when thyroid hormone (TH) reaches critical levels within cells and thus transcriptional output is dose-dependent. The binding of TH to TRs de-represses HDAC corepressor complexes and activates transcription. It is possible that regeneration-specific, signaling mechanisms may act to de-repress HDAC corepressor complexes during regeneration to regulate transcription temporally and spatially. In support of this hypothesis, we note that many of the genes that were expressed differently in response to romidepsin treatment were also identified in an experiment that used a chemical inhibitor to block WNT-ligand secretion^23^. Further temporal and spatial analyses of gene expression and regulation will be needed to rigorously test this hypothesis.

## Methods

### Animal procedures

The use of pre-feeding stage axolotls does not require a protocol approved by the Institutional Animal Care and Use Committee (IACUC) at University of Kentucky, however embryos used in this study were treated according to the same ethical standards that apply to feeding axolotls. Some of the axolotl embryos were reared to feeding stages (>12 dph at 17–18 C) to image larval tail anatomy. These individuals were fed brine shrimp and cared for using standard axolotl husbandry protocols approved under IACUC protocol 2017–2580. Embryos (RRID:AGSC_100E) were obtained from the Ambystoma Genetic Stock Center (RRID:SCR_006372).

### Chemical Screen of Epigenetic Compounds

The amphibian tail amputation assay was described previously^23^. Briefly, developmental stage 42^38^ axolotl embryos were manually hatched by removing the egg jelly and membrane, anesthetized in 0.02% benzocaine, and administered tail amputations with a razor blade to remove 2 mm (~20% of the body length) of the distal tail tip. Axolotl embryos were then distributed into microtiter plates containing epigenetic targeting compounds from two libraries: (1) the Structural Genomics Consortium epigenetic chemical panel (N = 22); (2) the Selleckchem epigenetics compound library (N = 151). Chemicals were dissolved in DMSO and diluted to a stock concentration of 10 mM (0.1% DMSO). The initial screen tested 4 replicate embryos per chemical at 10 μM. All chemicals that inhibited tail regeneration were tested again at 10 μM and chemicals were considered hits if both tests showed inhibition. Some chemicals that were not inhibitory at 10 μm were re-tested and found to be inhibitory at 20 μM. Embryos were imaged at the time of amputation and 7 dpa. Tail and spinal cord measurements were tested statistically using Student’s t-test. Embryo survival and distal tail shape were used to classify chemicals as toxic, inhibitory, or having no effect on tail regeneration.

### Microarray analysis of romidepsin and belinostat

Three microarray experiments were performed. In the first experiment, 336 embryos were administered tail amputations (2 mm removed from distal tip with a razor blade) and placed into microtiter plates containing rearing water (40% modified Holtfreter’s Solution) and 0.1% DMSO (controls) or rearing water with 10 μm romidepsin or 10 μm belinostat. Exactly 1 mm of the distal tail tip was removed from 24 embryos immediately after tail amputation to obtain Day 0 samples. Tissues from 8 embryos were pooled into a 1.5 ml tubes with 0.5 ml of RNA-later (Qiagen) to obtain three replicate pools. This tissue sampling and pooling procedure was used to create replicate pools of tissue for 12 hpa, 24 hpa, 48 hpa, and 72 hpa. Four replicates were processed for all but 12 hpa control (3 replicates) and 12 hpa belinostat (2 replicates). In the second microarray experiment, the same tissue sampling design was followed to generate 3 replicates for 0–3 hr romidepsin-treated and control embryos for each of 2 time points (3 and 6 hpa). In the third experiment, the same tissue sampling design was followed to generate 3 replicates for 0–1 mpa romidepsin-treated and control embryos at 3 hpa.

The tissue samples were maintained at 4 C in RNA later prior to RNA isolation using first the Trizol method and then a Qiagen minikit with on-the-column DNAse treatment of DNA. Microarray hybridization using an Ambystoma Affymetrix array^39^ was performed by the University of Kentucky Microarray Core Facility. The raw microarray data (.CEL files) were deposited in the GEO database (accession number GSE118515). GeneChips were normalized using the *affy* R package^40^ to accomplish robust multichip averaging (RMA)^41^. Differential expression analysis was conducted using the *limma* R package^42^. RMA normalized signal intensity values were fit to a linear model and empirical Bayes smoothing applied to standard errors. Moderated *t*-tests were performed separately for each time point to identify probe sets that yielded significantly different average expression values as a function of treatment. These lists were further filtered using a false discovery rate of α = 0.05 and by requiring a 1.5-fold difference between treatment and control means. Hierarchical clustering of significant genes was performed using Pearson correlation as the distance metric. Optimal k parameters were selected by plotting the within-cluster sum of squares by k, varying from 1 through 10; the k aligning with the observed bend in the resulting plot was chosen as the k parameter. Heatmaps were constructed and visualized using *ComplexHeatmap*^43^. Graphs of probe signal intensities were visualized using *ggplot2*^44^. Correlation plots were constructed with *CorrPlot*^45^ and *ggplot2* using Pearson correlation.

### HDACi treatment of embryos

Axolotl embryos were administered tail amputations and placed into microtiter wells containing either rearing water and 0.1% DMSO, belinostat (10 μM) and 0.1% DMSO, or romidepsin (10 μM) and 0.1% DMSO. Embryos were initially treated for 7-days however shorter exposure times were also tested. For example, the briefest exposure time was for 1 mpa; after exposure, embryos were rinsed in 1 liter of rearing water before being placed into microtiter plates with 2 ml of rearing water. Embryos were imaged at 7 dpa and in some cases at later post-amputation times.

### Cell proliferation assay

Embryos at stage 42 were anesthetized and 0.5 ul of 8 mM EdU (EdU click-iT) was microinjected into the intraperitoneal cavity. Then, 2 mm of the distal tail tip was amputated and embryos were treated with either 10 μM romidepsin (n = 6) or 0.04% DMSO (n = 6). Subsequent steps of the EdU staining methodology followed the protocol reported in Baddar *et al*.^46^. All EdU labeled cells within 200 μ of the amputation plane were counted as well as cells staining positive for DAPI, after normalizing for tissue area. Student’s t-test was used to assess statistical significance and t-statistics with P < 0.05 were considered statistically significant.

## Supplementary information

SUPPLEMENTARY INFORMATION

Supplementary Table 1

Supplementary Table 2

Supplementary Table 3

Supplementary Table 4

Supplementary Table 5

Supplementary Table 6

## Data Availability

The raw microarray data (.CEL files) were deposited in the GEO database (accession number GSE118515).
