# Supplementary material for: HDAC Regulates Transcription at the Outset of Axolotl Tail Regeneration
Source: Sci Rep. 2019 May 1;9:6751. doi: 10.1038/s41598-019-43230-6 (PMC6494824; doi:10.1038/s41598-019-43230-6)
Supplement: Supplementary file 1 — SUPPLEMENTARY INFORMATION [file 41598_2019_43230_MOESM1_ESM.pdf]

HDAC Regulates Transcription at the Outset of Axolotl Tail Regeneration

S. Randal Voss[1], Larissa V. Ponomareva[2], Varun B. Dwaraka[1], Kaitlin E. Pardue[1], Nour W. Al Haj Baddar[1], A. Katherine Rodgers[1], M. Ryan Woodcock[1,3], Qingchao Qiu[1], Anne Crowner[1], Dana Blichmann[1], Shivam Khatri[1], Jon S. Thorson[2]

[1]Department of Neuroscience, Spinal Cord and Brain Injury Research Center, and Ambystoma Genetic Stock Center, University of Kentucky, Lexington, KY 40506

[2]College of Pharmacy and Center for Pharmaceutical Research and Innovation, University of Kentucky, Lexington, KY 40536

[3]Department of Biology, Keene State College, Keene, NH 03431

SUPPLEMENTARY INFORMATION

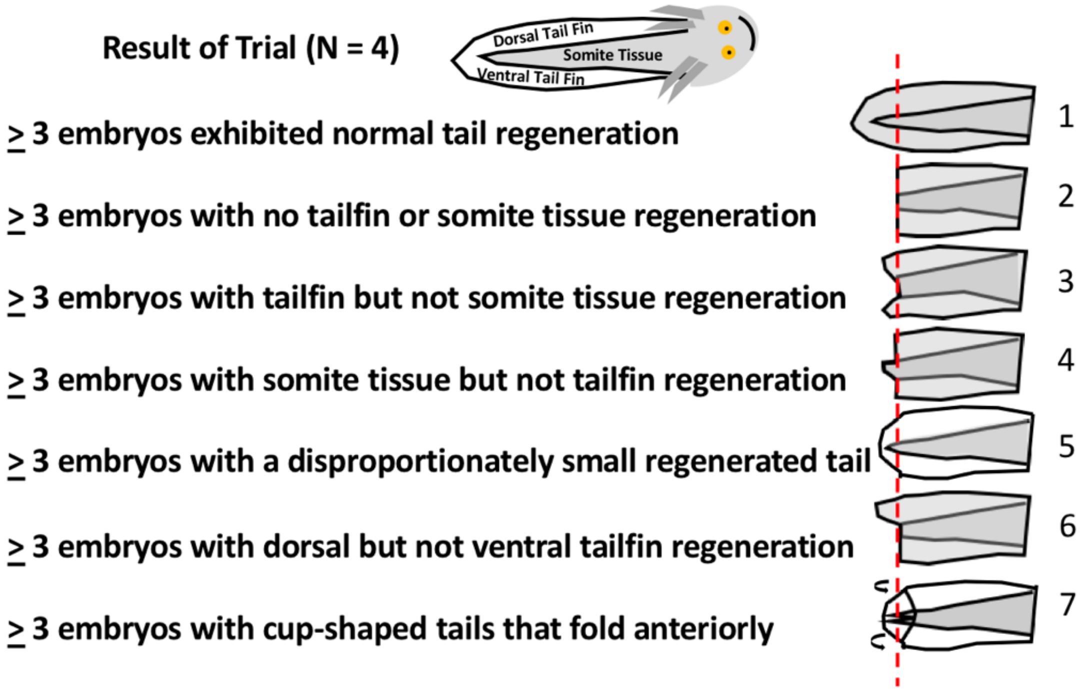

Supplemental File 1. Criteria that were used to differentiate a regenerative outcome (Classification 1) from inhibitory regenerative outcomes (Classifications 2-6) that were observed.

Supplementary Table 1. Chemicals from the Selleckchem and SGC libraries that were screened and the screening result.

Supplementary Table 2. Genes that were identified as differentially expressed between HDACi treated (romidepsin and belinostat) and control embryos. Log2 fold difference values (Col C-J) and p-values (Col K-R) are shown. Column S shows the Hierarchical Cluster ID for each gene (see Figure 2).

Supplementary Table 3. Experiments that were performed to determine the dose and delivery time for romidepsin inhibition of tail regeneration. 6-12 embryos were tested in each experiment. hpa = hours post amputation, mpa = minutes post amputation.

Supplementary Table 4. Genes that were identified as differentially expressed between romidepsin-treated and control embryos. Log2 fold difference values (Col C, D) and p-values (Col E, F) are shown. Column G shows the Hierarchical Cluster ID for each gene (see Figure 4).

Supplementary Table 5. Significantly enriched gene ontology terms for genes that were expressed more highly in romidepsin-treated versus control embryos at 3 and 6 hpa.

Supplementary Table 6. Significantly enriched gene ontology terms for genes that were expressed more highly in control versus romidepsin-treated embryos at 3 and 6 hpa.
